# Supplementary material for: APOBEC3A is the predominant global editor of cytosines in human mRNAs and in single-strand RNA viruses
Source: bioRxiv. 2026 Feb 12:2026.02.10.705151. Preprint. [Version 1] doi: 10.64898/2026.02.10.705151 (PMC12918798; doi:10.64898/2026.02.10.705151)
Supplement: Supplement 2 [file NIHPP2026.02.10.705151v1-supplement-2.pdf]

Supplementary Information

Supplementary Figure

S1 Figure. Mutagenesis by single-domain and by full-size double-domain (dd) APOBEC3G in yeast with wild type *RFA1* and hypomorph *rfa1-t33* alleles.

CAN1-R mutation rates measured in *RFA1-WT* and *rfa1-t33* strains, carrying either empty vector (EV), single domain APOBEC3G (A3G) or double-domain APOBEC3G (ddA3G) plasmids. Data for EV and single-domain A3G were taken from (DENNEN *et al.* 2024). Shown are median values for mutation rates measured in 6 independent cultures and 95% confidence intervals for the medians. Statistically significant difference with $p < 0.05$ of the one-tailed Mann–Whitney test showing mutagenic activity of ddA3G in rfa1-t33 background is indicated by brackets. Source data and statistical analyses for all pairwise comparisons can be found in S1 Table.

Supplementary Tables

S1 Table. Comparison of mutagenesis in CAN1 gene caused by single-domain APOBEC3G (A3G) and double domain ddAPOBEC3G in RFA1 and rfa1-t33 yeast strains.

S2 Table. Lists of files with DNA mutation and RNA editing calls analyzed in this study Table contains only file lists. Files with DNA mutation and RNA editing lists can be found in: https://doi.org/10.5281/zenodo.18079216

bioRxiv preprint doi: https://doi.org/10.64898/2026.02.10.705151; this version posted February 12, 2026. The copyright holder for this preprint (which was not certified by peer review) is the author/funder. This article is a US Government work. It is not subject to copyright under 17 USC 105 and is also made available for use under a CC0 license.

S3 Table. Values of motif enrichments used on graphs shown on Figures 1-6

S4 Table. DNA motif-centered statistical analyses using 96 trinucleotide-centered mutation motifs

S5 Table. Comparison between odds-ratios of different motifs and the same motif in different cohorts

S6 Table. DNA motif-centered statistical analyses using 192 trinucleotide-centered mutation motifs

S7 Table. Motif-centered statistical analyses using 192 trinucleotide-centered RNA editing motifs

Sequences are shown in DNA format (T instead of U) to maintain compatibility with other outputs of mutation signatures

S8 Table. Secondary structure preferences of C to U mRNA edits in BT-474 human breast cancer cell line transfected with APOBEC3A or APOBEC3 vector

S9 Table. List of accession numbers and publications associated with poliovirus genomes analyzed in this paper

S10 Table. Yeast and human cell lines used in this study

S11 Table. Detailed documentation of the CLC Genomics Workbench workflow used for calling DNA mutations in bulk yeast cultures expressing strong APOBECs, A3A and A3B

S12 Table. Detailed documentation of the CLC Genomics Workbench workflow used for calling DNA mutations in can1 isolated from cultures expressing weaker APOBECs, A1, A3C, ddA3G

Figure S1

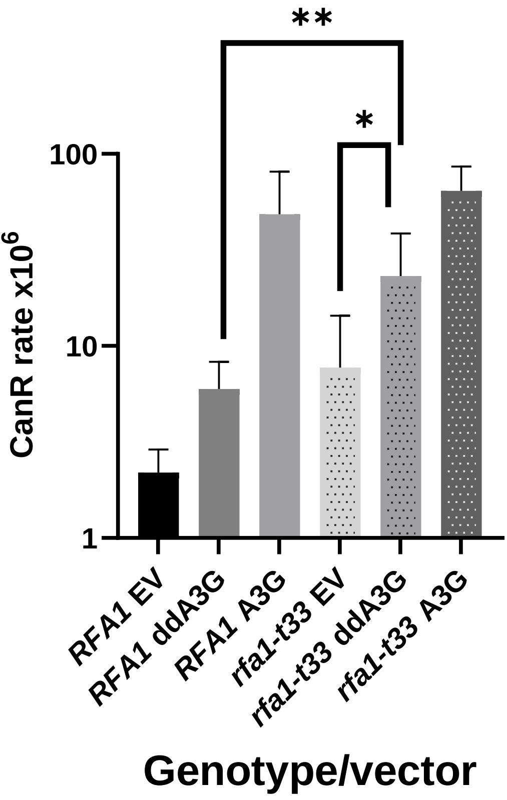
**
*
100
10
1
CanR rate x10^6
RFA1 EV
RFA1 ddA3G
RFA1 A3G
rfa1-t33 EV
rfa1-t33 ddA3G
rfa1-t33 A3G
Genotype/vector
